# A Quantitative Multiparametric MRI Analysis Platform for Estimation of Robust Imaging Biomarkers in Clinical Oncology

**DOI:** 10.3390/tomography9060161

**Published:** 2023-11-03

**Authors:** Eve LoCastro, Ramesh Paudyal, Amaresha Shridhar Konar, Peter S. LaViolette, Oguz Akin, Vaios Hatzoglou, Alvin C. Goh, Bernard H. Bochner, Jonathan Rosenberg, Richard J. Wong, Nancy Y. Lee, Lawrence H. Schwartz, Amita Shukla-Dave

**Affiliations:** 1 Department of Medical Physics, Memorial Sloan Kettering Cancer Center, New York, NY 10065, USA; locastre@mskcc.org (E.L.); paudyalr@mskcc.org (R.P.); amareshks@gmail.com (A.S.K.); 2 Department of Radiology, Medical College of Wisconsin, Milwaukee, WI 53226, USA; plaviole@mcw.edu; 3 Department of Radiology, Memorial Sloan Kettering Cancer Center, New York, NY 10065, USA; akino@mskcc.org (O.A.); hatzoglv@mskcc.org (V.H.); schwartzl@mskcc.org (L.H.S.); 4 Department of Surgery, Memorial Sloan Kettering Cancer Center, New York, NY 10065, USA; goha@mskcc.org (A.C.G.); bochnerb@mskcc.org (B.H.B.); wongr@mskcc.org (R.J.W.); 5 Department of Medicine, Memorial Sloan Kettering Cancer Center, New York, NY 10065, USA; rosenbj1@mskcc.org; 6 Department of Radiation Oncology, Memorial Sloan Kettering Cancer Center, New York, NY 10065, USA; leen2@mskcc.org

**Keywords:** multiparametric MRI, dynamic contrast-enhanced MRI, diffusion-weighted MRI, optimal model mapping, cancer, oncology, quantitative imaging biomarkers

## Abstract

There is a need to develop user-friendly imaging tools estimating robust quantitative biomarkers (QIBs) from multiparametric (mp)MRI for clinical applications in oncology. Quantitative metrics derived from (mp)MRI can monitor and predict early responses to treatment, often prior to anatomical changes. We have developed a vendor-agnostic, flexible, and user-friendly MATLAB-based toolkit, MRI-Quantitative Analysis and Multiparametric Evaluation Routines (“MRI-QAMPER”, current release v3.0), for the estimation of quantitative metrics from dynamic contrast-enhanced (DCE) and multi-b value diffusion-weighted (DW) MR and MR relaxometry. MRI-QAMPER’s functionality includes generating numerical parametric maps from these methods reflecting tumor permeability, cellularity, and tissue morphology. MRI-QAMPER routines were validated using digital reference objects (DROs) for DCE and DW MRI, serving as initial approval stages in the National Cancer Institute Quantitative Imaging Network (NCI/QIN) software benchmark. MRI-QAMPER has participated in DCE and DW MRI Collaborative Challenge Projects (CCPs), which are key technical stages in the NCI/QIN benchmark. In a DCE CCP, QAMPER presented the best repeatability coefficient (RC = 0.56) across test–retest brain metastasis data, out of ten participating DCE software packages. In a DW CCP, QAMPER ranked among the top five (out of fourteen) tools with the highest area under the curve (AUC) for prostate cancer detection. This platform can seamlessly process mpMRI data from brain, head and neck, thyroid, prostate, pancreas, and bladder cancer. MRI-QAMPER prospectively analyzes dose de-escalation trial data for oropharyngeal cancer, which has earned it advanced NCI/QIN approval for expanded usage and applications in wider clinical trials.

## 1. Introduction

Technical developments in MRI hardware and data acquisition have given us unique insights into the biology of normal tissue and complex diseases [1,2]. The acquired anatomic and physiological MR images can be used for qualitative evaluation [3,4] and quantitative measurement of model-based imaging metrics from multiparametric (mp)MRI [5,6,7]. For example, quantitative imaging biomarkers (QIBs) derived from dynamic contrast-enhanced (DCE)-MRI, multi-b value diffusion-weighted (DW)-MRI, and MR relaxometry are surrogates of tumor perfusion, cellularity, and tissue morphology [8]. Furthermore, qualitative and quantitative analyses of mpMRI data have shown promise for tumor detection, assessment of treatment response, and outcomes in clinical oncology [4,7,8,9,10].

Some challenges to the clinical application of mpMRI include robust data acquisition, analysis techniques, and standardized guidelines for interpreting the results [11]. The limitations also include the limited availability of comprehensive mpMRI analysis tools and the reporting standards of the robustly derived QIBs [7,8]. For this purpose, the Quantitative Imaging Network (QIN) of the National Cancer Institute (NCI) has developed a five-level benchmark of criteria used to evaluate the performance and clinical readiness of aspiring software tools to provide QIB analysis for prospective clinical treatment and research [12]. The benchmarks range from initial testing against digital reference objects (DRO), participation in collaborative challenge projects (CCPs) with test–retest results, and demonstration of its usage in clinical trials [12]. Digital reference objects (DROs) are integral to developing and standardizing data analysis routines [13]. DROs offer a known ground truth and are artificially generated images designed to mimic acquired data [14]. The advantages of DROs include adding an explicit amount of random noise that distorts the generated signal, which tests an algorithm’s performance to see whether it can recover the known ground truth despite inconsistencies in image quality that are typically present in real-world images [7]. The scope of available QIN-approved tools spans multiple imaging modalities (CT, PET, and MRI) with a range of functionalities, including quality assurance tools, quantitative parametric mapping, and auto-segmentation routines [15]. Mature software tools in the QIN catalog must be proven to work consistently with DROs and CCPs.

Therefore, we have developed a vendor-agnostic, full-featured, and user-friendly MATLAB-based toolkit, Quantitative Analysis and Multiparametric Evaluation Routines for MRI (“MRI-QAMPER”, current release v3.0). QAMPER performs the extraction of robust QIBs from mpMRI data, and we have progressively worked to validate results through QIN software benchmarks [12]. QAMPER’s principal functionality is to generate parametric maps reflecting tumor permeability, cellularity, and tissue morphology. The mpMRI data from the brain [16], head and neck [17], thyroid [18,19], prostate [20], pancreas [21], and bladder (under review) have been seamlessly analyzed with QAMPER. Parametric maps obtained from quantitative mpMRI images can reveal spatial heterogeneity within tumors and provide numerical values for the underlying physiology.

Major MRI vendors have the capability to perform semi-quantitative DCE analysis and monoexponential DW modeling at the scanner. There are open-source resources with models for DCE and/or DW and/or MR relaxometry analysis [22,23]. QAMPER is a one-stop shop for converting and analyzing mpMRI, which can be directly implemented in MATLAB without the need for users to compile software binaries. When considering quantitative imaging metrics from DCE and DW MRI data, most studies apply a single data-fitting model to the whole tumor. However, no single model is sufficient to capture all the physiological characteristics of a tumor [24,25]. The optimal model mapping (OMM) method implemented in MRI-QAMPER for DCE [19] and DW MRI [25] performs voxel-wise analysis using multiple data fitting algorithms within tumor regions of interest based on a statistical approach. The OMM selection method serves to identify the best model to describe the data and to aid in depicting and understanding tumor heterogeneity.

In the Methods section of this article, we will explain in detail how we implemented our platform for mpMRI data analysis, from DICOM import and conversion to numerical parameter map generation and nested model selection. The Results section summarizes the NCI/QIN software benchmark validation steps explored through DRO and QIN CCP avenues.

## 2. Materials and Methods

MRI-QAMPER is currently available as a MATLAB-based toolkit (The Mathworks, Natick, MA, United States). The software imports DICOM images from major MRI vendors and different field-strength MRI scanners for post-processing of DCE MRI, multi-b-value DW MRI, $T_{1}$ mapping with multi-flip angle, and $T_{2}$ mapping with multi-echo MR relaxometry. It includes organ-specific presets and boundary conditions, allowing user-customized parameters for each non-linear fitting model of mpMRI data. MRI-QAMPER is approved by NCI/QIN and has currently earned Level 5 “Clinical Benchmark” status for use in clinical trials [12].

**Figure 1:**
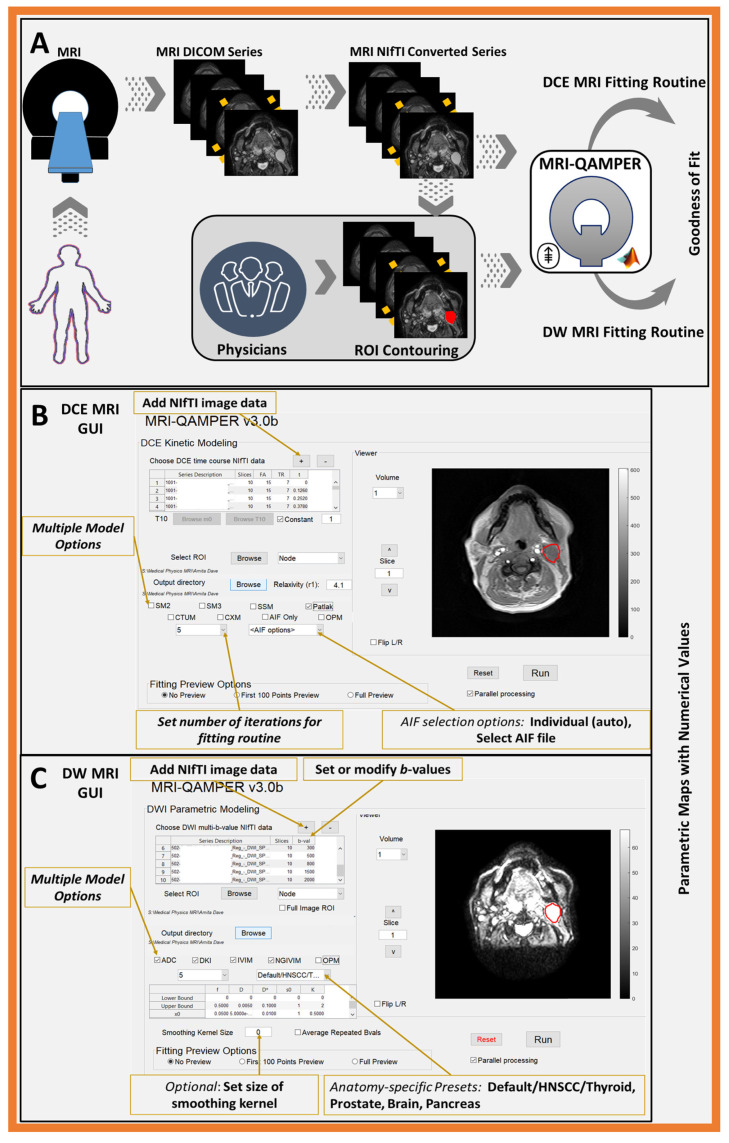
Workflow of MRI-QAMPER v3.0: (**A**) Images are acquired from MRI scanner and converted from DICOM to NIfTI. Skilled physician or planner contours ROI on image. The NIfTI MR images and ROI are loaded into the MRI-QAMPER GUI. (**B**) View of the MRI-QAMPER DCE GUI, with preview of patient image with ROI overlaid. Interface provides options for selecting multiple DCE routine for analysis, parameter bounds, option for OMM and AIF. (**C**) View of the MRI-QAMPER DW GUI, with patient and ROI preview. Interface provides options for selecting multiple DW routines, parameter bounds, manual toggling/editing of b-values and OMM option.

**Figure 2:**
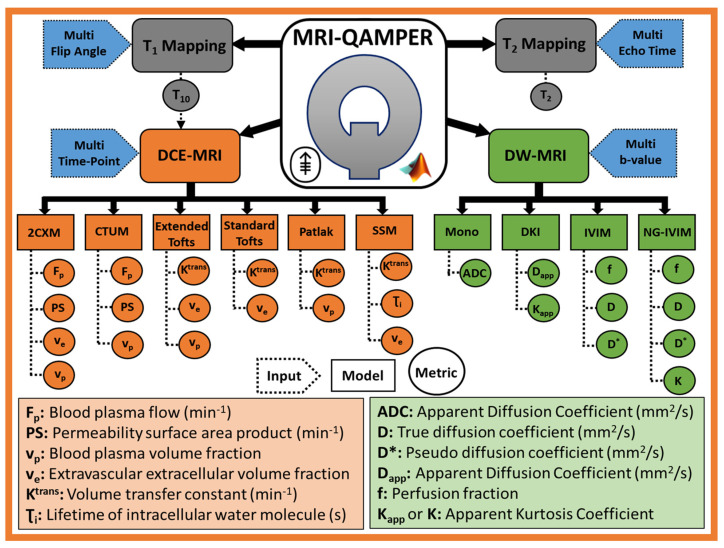
Schematic of image processing routines included with MRI-QAMPER v3.0. The software provides methods for: multi-flip angle $T_{1}$ mapping, multi-echo $T_{2}$ relaxometry, multi-compartmental methods for DCE, and fitting for multi-b-value DW imaging.

Figure 1 shows an overview of the MRI-QAMPER workflow. The graphical user interface offers an intuitive, step-by-step process to streamline analysis. The software is vendor-agnostic and will analyze mpMR images from the brain to the pelvis in clinical studies. It can be easily modified for other organs or preclinical data. QAMPER works natively with NIfTI image files [26] and includes an interface to convert DICOM images and import relevant image header acquisition metadata (i.e., flip angle (FA), repetition time (TR), number of phases, and b-values, as necessary). Regions of Interest (ROIs) label masks are saved in NIfTI imaging format in the same space and resolution as the MRI sequence for processing. Our group employs ITK-SNAP [27] to contour and save compatible NIfTI files for use with MRI-QAMPER, and to review result maps. Figure 2 is a schematic summary of the variety of models available for processing DCE, DW, $T_{1}$, and $T_{2}$ MRI data. QAMPER’s analyses range from commonly available data fitting routines [28] to more advanced data modeling algorithms [5,24,25,29,30] and MR relaxometry analysis methods, which are detailed as follows.

### 2.1. DCE MRI Pharmacokinetic Modeling

DCE MRI time course analysis focuses on extravasation and transport of contrast agent (CA) in tissue, particularly their distribution within and elimination from the body [9]. $T_{1}$-weighted DCE imaging involves a series of data acquisition before, during, and after intravenous injection of a Gadolinium-based CA [28]. $T_{1}$ mapping is performed with multiple FAs for the estimation of pre-contrast $T_{1}$ (ie, $T_{10}$) [31,32] needed for Equation (1). The spoiled gradient recalled (SPGR) acqusition Equation used for $T_{10}$ fitting is provided in Appendix A (Equation (A2)).

The change in water proton relaxation rate (i.e., ${\Delta R}_{1}(t)={(R}_{1}(t)-R_{10})$ [s^−1^]) due to CA relaxivity, $r_{1}$ [(mM)^−1^s^−1^], is linearly related to the tissue CA concentration, $C_{t}$ (mM),

(1) $${R_{1}\left(t\right)=R_{10}{+r}_{1}C}_{t}\left(t\right)\;\to {\Delta R}_{1}\left(t\right)=r_{1}C_{t}(t),$$

where R_10_ is the pre-contrast longitudinal relaxation rate.

The signal in the DCE images is converted to tissue CA concentration via longitudinal $T_{1}$-relaxation [31], requiring measurement of the pre-contrast R_10_ (=1/$T_{10}$) and arterial input function (AIF) from the major artery within the field of view (FOV). DCE pharmacokinetic analysis can be performed to estimate physiological parameters via compartmental models ranging from complex to simple, providing plasma (blood) flow ($F_{p}$), permeability surface area product (PS), volume fractions of blood plasma ($v_{p}$), and extracellular extravascular space (EES) ($v_{e}$).

Figure 2 shows the various DCE pharmacokinetic models that can be used to analyze data where the application of the optimal model selection depends on tumor heterogeneity. The most general two-compartment exchange model (2CXM: $F_{p}$, PS, $v_{e}$, and $v_{p}$) [5] reduces to nested simpler models under special approximation, including the compartmental tissue uptake model (CTUM $F_{p}$, PS, and $v_{p}$) [5], extended Tofts model (ETM: $K^{trans}$, $v_{e}$, and $v_{p}$) [28], Tofts Model (TM: $K^{trans}$ and $v_{e}$) [28], and Patlak model (PM: $K^{trans}$ and $v_{p}$) [33]. These models assume an infinitely fast water exchange between the tissue compartments, called fast water exchange limit (FXL). On the other hand, the shutter speed model (SSM) model (also referred to as fast exchange regime (FXR)) describes the finite rate of water exchange across the cell membrane between intracellular space (ICS) and EES [34], providing estimates of the mean lifetime of the water molecule in intracellular space ($\tau_{i}$) in addition to $K^{trans}$ and $v_{e}$. The comprehensive list of DCE MRI routines available in QAMPER includes 2CXM, CTUM, ETM, TM, PM, and SSM (Figure 2). Herein, we describe the commonly used Tofts models.

#### 2.1.1. Extended Tofts Model (ETM)

ETM assumes a two-compartment model (vascular space and EES) expression for modeling $C_{t}(t)$ is given by Equation (2), and standard TM is obtained assuming a weakly vascularized ($v_{p}$→0) (Equation (3)).

(2) $$C_{t}(t)=K^{trans}e^{-k_{ep}}⊗C_{p}\left(t\right)+{v_{p}C}_{p}(t)$$

(3) $$C_{t}(t)=K^{trans}e^{-k_{ep}}⊗C_{p}\left(t\right)$$

where the ⊗ operator denotes the convolution function. $C_{p}$ and $C_{t}$ are the plasma space and tissue CA concentrations, respectively. $k_{ep}$ is the backward flux from the EES to vascular space.

#### 2.1.2. Patlak Model (PM)

The two-parameter Patlak model ignores back flux to the vascular space from EES. The extended Patlak model is equivalent to the ETM [33].

(4) $$C_{t}(t)=K^{trans}⊗C_{p}\left(t\right)+{v_{p}C}_{p}(t)$$

#### 2.1.3. Fast Exchange Regime (FXR) or Shutter Speed Model (SSM)

In biological tissue, water protons reside in three compartments: intravascular space, EES, and ICS [35]. Equilibrium water exchange kinetics occur between these compartments. Two- and three-site two-water exchange models have been developed to account for the water exchange kinetics in estimates of $T_{1}$ relaxation [36]. FXR is a two-compartment model that accounts for equilibrium water exchange across cell membranes between intracellular space and EES [34]. FXR is formulated in Equation (5), in terms of nonlinear $R_{1}$ (${\equiv 1/T}_{1}$_,_) incorporating EES CA concentration ($C_{e}\left(t\right)=C_{t}\left(t\right)/v_{e}$) from Equation (3) into Equation (5), rather than using the linear relationship between the change in $R_{1}$ (Δ$R_{1}$) with *C*_t_ (Equation (1)). The equation for FXR is given by

(5) $$R_{1t}\left(t\right)=\frac{1}{2}\left[\left(R_{1i}+k_{ie}{+R_{10e}+r_{1}\;C_{e}\left(t\right)+k}_{ei}\right)-\sqrt{{\left(R_{1i}+k_{ie}-R_{10e}-r_{1}\;C_{e}\left(t\right)-k_{ei}\right)}^{2}+4{\;k}_{ie}k_{ei}}\right]$$

where $R_{1i}$ and $R_{10e}$ are pre-contrast $R_{1}$ for ICS and EES; $k_{ie}$ in the inverse of mean lifetime of intracellular water protons, $\tau_{i}$=1/$k_{ie}$; and $k_{ie}$ is the rate of water exchange from ICS to EES (vice versa for $k_{ei}$).

Detailed equations for signal-to-CA conversion, CTUM, and 2CXM models are provided in the Appendix A.

#### 2.1.4. Arterial Input Function (AIF) Selection

QAMPER provides options to perform DCE analysis using an AIF of the user’s choice. This may be a curated or population-based AIF [37] or extraction of an individual-based AIF using QAMPER’s automated method. Automated AIF detection is performed by identifying the major artery in the image field of view using time-wise cross-correlation analysis against an idealized reference function for each voxel in the DCE volume [38]. The contrast-enhanced image signal intensity curve is converted to $C_{p}\left(t\right)$ [31], which can then be used as the impulse function for compartmental DCE models.

### 2.2. DW MRI Data Modeling

Quantitative DW MRI measures the Brownian motion of water molecules at a cellular level. Multi-b-value acquisitions at low b-values (b ≤ 0–100 s/mm^2^) and intermediate to high b-values (b ≥ 100–2000 s/mm^2^) exhibit two distinct curvature signals. The standard monoexponential model calculates the apparent diffusion coefficient (ADC, Equation (6)), reflecting tumor cellularity, from imaging with at least ≥ 2 b-values using a straightforward linear regression fitting [39]. ADC is a composite metric accounting for molecular diffusion and microcapillary perfusion. LeBihan formulated a biexponential model (intravoxel incoherent motion (IVIM)), fitting multi-b-value signal with estimated metrics of the capillary network, i.e., pseudo-diffusion coefficient ($D^{*}$), perfusion fraction (f), and tissue true diffusion coefficient (D) (Equation (8)) [29]. IVIM estimation of f provides insight into vascular dynamics without CA injection. ADC and IVIM models assume a Gaussian probability distribution function for the displacement of the water molecules. The presence of underlying tissue microstructures in a tumor can alter the distribution of water diffusion from Gaussian to non-Gaussian (NG). Diffusion kurtosis imaging (DKI) (Equation (7)) was introduced to capture NG effects by expanding the DW signal to second-order higher b-values [30], represented by apparent kurtosis coefficient ($K_{app}$), a surrogate QIB of tissue microstructure, in addition to the apparent diffusion coefficient ($D_{app}$). NG-IVIM describes simultaneous perfusion and restricted diffusion by incorporating microstructure, K, the signal deviation from Gaussianity [25,29], providing estimates of the f, $D^{*}$, D, and K. Figure 2 shows QAMPER’s available DW models, which can be used to analyze data and to identify the optimal model based on tumor heterogeneity. The DW MRI model equations are as follows:

2.2.1. Monoexponential model [39]:

(6) $$S_{b}=S_{0}e^{-b\times ADC}$$

where $S_{b}$ and $S_{0}$ are signal intensities with and without diffusion-weighting factor b (s/mm^2^), respectively.

2.2.2. DKI model [30]:

(7) $$S=S_{0}\;e^{-b\times D_{app}+\frac{1}{6}K_{app}{\left(b\times D_{app}\right)}^{2}}$$

2.2.3. Intravoxel incoherent motion (IVIM) model [29]:

(8) $$S=S_{0}(fe^{-b\times D^{*})}+\left(1-f\right)e^{-b\times D})$$

2.2.4 Non-Gaussian intravoxel incoherent motion (NG-IVIM) model [25]:

(9) $$S=S_{0}\;{({fe}^{-b\times D^{*})}+\left(1-f\right)e}^{-b\times D+\frac{1}{6}K{\left(b\times D\right)}^{2}})$$

### 2.3. $T_{1}$ and $T_{2}$ Relaxometry

$T_{1}$ and $T_{2}$ relaxometry measurements assess the water content fraction and vascular morphology through multiple acquisitions of varying imaging parameters [40]. For quantification of $T_{1}$ and $T_{2}$ values, the standard approaches are inversion recovery (IR) with different inversion time (TI) values and multiple single spin-echo (SE) methods with varying echo time (TE) values. Various methods have been developed to quantify $T_{1}$ and $T_{2}$ metric values within a clinically feasible timeframe [32,41,42,43]. Multi-echo $T_{2}$ mapping [44] can be tailored appropriately for different anatomical regions and physiological conditions.

Variable flip angle $T_{1}$ measurement allows $T_{1}$ value estimation in a clinically feasible time using multiple spoiled gradient echo acquisitions with different FA [32]. The Equation is given in Appendix A (A2).

A multi-echo spin-echo sequence is commonly used for $T_{2}$ mapping, and the Equation is given by [44].

(10) $$S=S_{0\;}e^{-\frac{TE}{T_{2}}}$$

where TE is the echo time.

### 2.4. Optimal Model Mapping (OMM)

Model selection techniques for DCE or DW MRI data fitting refer to identifying the best model that more closely fits the data set for each voxel [24,25,31,45]. Heterogeneity within solid tumors and surrounding tissue, irregular levels of vasculature development, and leakiness are represented by different compartment models. The data acquisition methods and underlying tumor physiology need to be considered while applying OMM. The F-Statistic (F-test) [46], R-square (R^2^) [31], chi-square (χ^2^) [47], corrected Akaike information criteria (AICc) [19,48,49,50], and Bayesian information criteria (BIC) [25,51] have been used to select the model that most accurately reflects the tumor physiology and can be used to quantify values of imaging biomarkers in each voxel.

### 2.5. Imaging Formats and Conversion

QAMPER utilizes the dcm2niix conversion application to import DICOM images into NIfTI format for processing. The software readily accepts NIfTI images of ROI masks in matching coordinate space to the parent image, and generated parametric maps are output as NIfTI files. The output mapping data are simultaneously saved as MATLAB struct arrays in MAT files, plus additional metrics to assess the quality of fitted output: AIC [48], BIC [51], and R^2^. In addition to MATLAB, two open-source external dependencies are currently required to run MRI-QAMPER: dcm2niix [26] and NIfTI Toolbox for MATLAB.

## 3. Results

**Figure 3:**
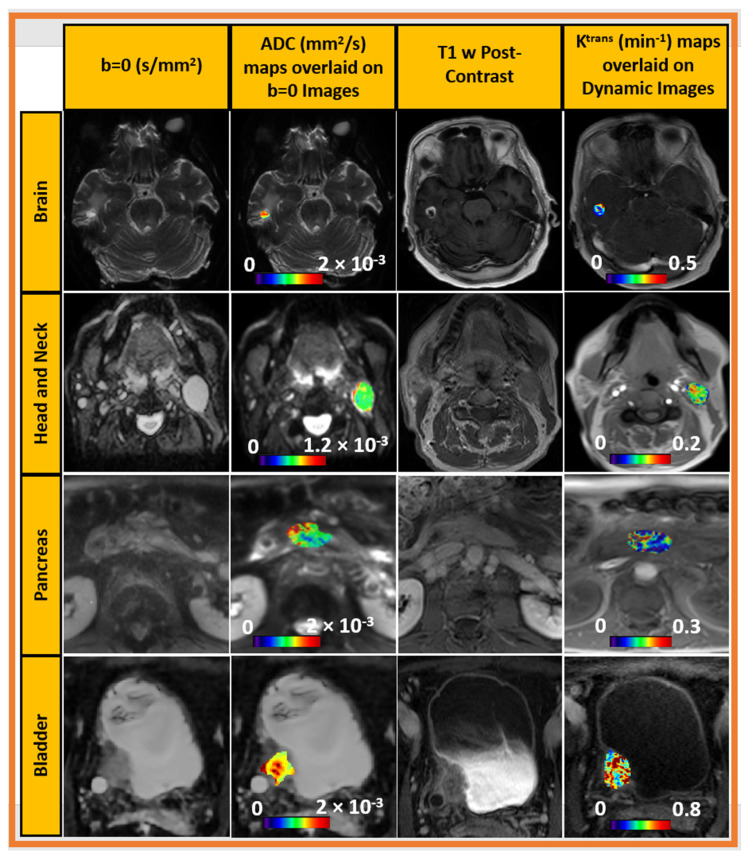
Representative output of MRI-QAMPER v3.0: input images (DW b = 0, $T_{1}$-weighted base image) and output quantitative parametric maps (ADC, $K^{trans}$), computed for images in brain, head and neck, pancreas and bladder. Visualization of parametric map overlay was created with MRIcron (v1.0.20190902) software.

At our center, QAMPER has been used to analyze mpMRI data, which resulted in peer-reviewed articles on outcomes in various organs, such as the brain [16], head and neck [17], thyroid [18,19], prostate [20], pancreas [21], and bladder (article under review) (Figure 3). The software was evaluated using DROs and participated in CCPs and in clinical trials.

### 3.1. QAMPER QIN Software Validation: DROs and CCPs

The initial performance of the MRI-QAMPER routines was validated using well-established digital reference objects (DROs) for DCE and DW MRI, serving as early approval stages in the QIN software benchmark [12]. MRI-QAMPER has participated in DCE and DW QIN CCPs: (i) MR Relaxometry Brain OSIPI DCE Challenge (in press) led by Dr. Anahita Fathi Kazerooni (UPENN and CHOP) and (ii) Multi b-Value Prostate Challenge [20] led by Dr. Peter S. LaViolette (MCW). The software is approved for clinical trials and is routinely used for mpMRI data analysis in an oropharyngeal cancer dose de-escalation clinical trial (#NCT03323463) at MSKCC [10].

### 3.2. DCE MRI DRO (RSNA)

QAMPER DCE perfusion routines were tested using the DCE DRO from the Radiological Society of North America (RSNA) Quantitative Imaging Biomarkers Alliance (QIBA; noise-free, three-parameter, extended, https://sites.duke.edu/dblab/qibacontent/, accessed on 10 October 2023) [52], developed in the Barboriak Lab, Duke University [14]. The ETM, three-parameter fit, model was used to fit a synthetic data slice with 661 time points. The resulting $K^{trans}$ map from QAMPER showed excellent agreement with the ground truth.

### 3.3. DW MRI DRO (University of Michigan)

The diffusion DW MRI DRO developed by the Chenevert Lab, University of Michigan, was used to evaluate the performance of QAMPER routines for ADC, IVIM, and DKI [53]. This set of DROs was generated for advanced DW MRI clinical trial protocols. The generated DROs include simulated acquisition noise, DICOM scaling, and clinically relevant DW MRI parameter ranges for perfusion-fraction IVIM and DKI models. The resulting DW MRI-derived parametric maps showed excellent agreement with the ground truth.

### 3.4. Collaborative Challenge Projects

#### 3.4.1. DCE CCP (ISMRM, Open Science Initiative for Perfusion Imaging (OSIPI))

The OSIPI Task Force on DCE/DSC challenges was formed to benchmark perfusion quantification methods by route of organized community challenges (in press) to craft a systematic and controlled framework to benchmark and progress $K^{trans}$ calculation as a biomarker in brain tumors. This study reports results from the OSIPI-DCE challenge and highlights the high inter-software variability within $K^{trans}$ estimation. The OSIPI-DCE challenge assessed the performance of 10 participating software packages according to their ability to accurately calculate $K^{trans}$ from a set of generated synthetic data, repeatability of parameter calculation in test–retest scans of eight patients with glioblastoma, and reproducibility of the software from independent re-analysis of the data. QAMPER finished in the top four software packages out of ten at recapturing $K^{trans}$ values from the synthetic data. QAMPER presented the best repeatability coefficient across the test–retest data among all packages tested, with RC = 0.56.

#### 3.4.2. DW MRI CCP (QIN, MCW, Prostate)

A collaborative group organized by the NCI coordinated a multi-institutional study to explore how differences in DW fitting algorithms affect prostate cancer detection and how varying post-processing parameters differentiate regions of tumors by severity [20]. Results from 14 groups and software were verified against ground truth histological maps and pathologist-traced MRI annotations. The study aimed to measure the consistency of values in calculated metrics between sites and evaluate inter-site variability in performing a diagnostic task. Mono-exponential and kurtosis analyses were the most stable, presenting a low percent difference and high correlation coefficient, independent of site pairing permutations. The DW MRI study found that conventional diffusion models consistently differentiated prostate cancer from benign tissue. The results also indicated that post-processing decisions on DW MRI data could affect sensitivity and specificity when applied to radiological–pathological studies in prostate cancer. The parameters K, diffusion kurtosis (D_K_), and ADC showed the least percent difference among sites and the highest correlation. QAMPER’s ability to customize and set lower- and upper-threshold bounds was a feature that grouped it among the top five out of fourteen tools with the highest area under the curve (AUC) for prostate cancer detection [20].

### 3.5. DCE and DW MRI in Clinical Trial (Oropharyngeal Cancer)

At our center, MRI-QAMPER is routinely used to analyze mpMRI data obtained from oropharyngeal cancer patients enrolled in the dose de-escalation clinical trial (#NCT03323463) [10]. Pretreatment DCE MRI analyses confirmed a difference in perfusion and permeability between patients with residual disease and those with pathological complete response (pCR). In addition, in a small pilot study, DW MRI revealed changes in the ADC and relative K that were statistically significantly correlated with pCR at the time of surgery [10].

## 4. Discussion

Our in-house MATLAB toolbox was developed for the estimation of robust QIBs from mpMRI. DCE and DW MRI results obtained from MRI-QAMPER routines have been featured in numerous peer-reviewed publications [8,17,19,54]. The core software development has been supported by NIH funding and extended into an easily installable MATLAB package with a graphical interface and capability for conversion of DICOM files and extraction of imaging metadata. MRI-QAMPER’s ability to extract timing information from DCE MRI and b-values from DW MRI allows it to operate on MR images independent of the scanner vendor. The bounds for nonlinear fitting parameters can be user-set to be appropriate on a case-by-case basis, ensuring that results are physiologically relevant. The analysis routines are organ-independent due to the customizable parameters available in the MRI-QAMPER software and include several literature-based presets.

In oncological imaging, there is a need for proven quantitative imaging tools and the development of MRI QIBs for specific clinical endpoints [7,8,12]. Quantitative analysis of mpMRI data estimates parameters characterizing underlying tumor physiology. All major MRI vendors have tools for DCE MRI semi-quantitative analysis or Standard Tofts (e.g., NordicICE; Nordic Neuro Lab, Bergen, Norway). Currently, a selection of open-source tools exists for time-course compartmental models or basic Tofts analysis of DCE data (e.g., Madym [22], MITK-ModelFit [55], FireVoxel (https://firevoxel.org/, accessed on 10 October 2023), DCENET [56]). DW MRI tools for monoexponential ADC modeling are widely available on all major MRI vendor platforms. Many cutting-edge advanced analysis routines for bi-exponential or kurtosis imaging from other providers are still closed source, based on in-house code, limiting wider adoption [25]. Few tools offer a one-stop shop for multiple modalities. One comparable example is the C++-based Madym software (v4.15.2), which can analyze DCE, DWI (ADC, IVIM), and relaxometry ($T_{1}$ and $T_{2}$ mapping) [22], but does not have OMM or verification status offered by NCI/QIN Benchmarking. OMM is provided by the ROCKETSHIP platform [23] for DCE models, but there is no other similar offering for DW MRI.

**Figure 4:**
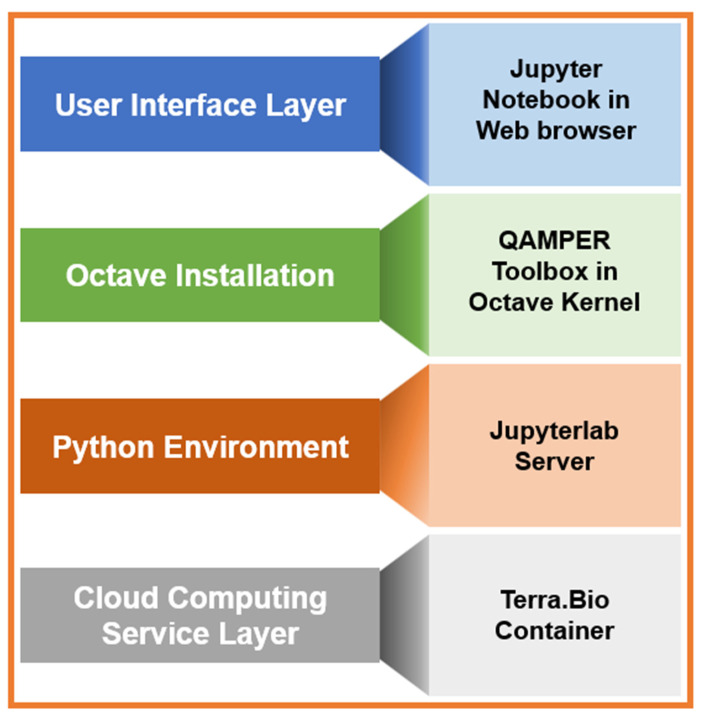
Proposed architecture schema for cloud implementation of MRI-QAMPER.

QAMPER is currently available through a material transfer agreement (MTA) with our center. Our group has experience with MR Fingerprinting (MRF) (refs: phantom, brain metastases [57,58]), which provides simultaneous $T_{1}$ and $T_{2}$ relaxometry measures in a clinically feasible amount of time. We plan to include the MRF reconstruction algorithm in our next implementation of the QAMPER platform. In the future, we plan to make MRI-QAMPER available via cloud architecture (Figure 4) [59].

## 5. Conclusions

Given the need to develop quantitative tools estimating robust imaging biomarkers for clinical applications in oncology, MRI-QAMPER (v3.0) is a standardized platform that can process mpMRI data seamlessly from the brain, head and neck, thyroid, prostate, pancreas, and bladder. Output of MRI-QAMPER routines has been validated using RSNA QIBA DROs for both DCE MRI (Barboriak Lab, Duke University) and DW MRI (Chenevert Lab, University of Michigan).

There are a number of platforms for quantitative MRI with either DCE and/or DW and/or MR relaxometry. QAMPER stands out due to its extensive features and vetting by NCI/QIN. In addition, none of the existing platforms have the non-Gaussian IVIM (NG-IVIM) DW MRI fitting routine in their packages, which was developed and implemented by our group [25].

ETM has been tested in the OSIPI Task Force DCE challenge (manuscript under review) to benchmark and progress $K^{trans}$ calculation as a diagnostic or prognostic biomarker in brain metastasis. Other DCE pharmacokinetic models, such as SSM, CTUM, and 2CXM, need to be validated through DRO and CCPs. DW MRI routines have been thoroughly vetted through DRO and CCP events, including the multi-b value prostate diffusion imaging challenge for prostate cancer detection led by Dr. Pete LaViolette at Medical College Wisconsin (MCW) [20]. The software has been distributed to NCI QIN collaborators to perform inter-site data validation testing and is available upon request through material transfer agreements (MTA).

## Data Availability

The data presented in this study will be provided upon reasonable request.

## Appendix A

DCE MRI pharmacokinetic analysis.

### Appendix A.1. Precontrast $T_{10}$ Fitting

A series of gradient-echo images are acquired using the variable flip angle (FA) method and fixed repetition time (TR) and echo time (TE). The signal intensity for the spoiled gradient recalled (SPGR) sequence is given by [31]

(A1) St=M0 sin⁡θe−TER2∗t1−e−TRR1t1−cos⁡θe−TRR1t

This can be written as follows to estimate pre-contrast longitudinal relaxation time, $T_{1}$, and equilibrium magnetization, $M_{0}$:

(A2) $$\frac{S(t)}{sin\left(\theta \right)}=E_{1}\frac{S(t)}{tan\left(\theta \right)}{+M}_{0}\left(1-E_{1}\right)$$

where $E_{1}=e^{-TRR_{1}\left(t\right)}$, $S\left(t\right)$ is the voxel signal intensity at time t, $M_{0}$ is the equilibrium magnetization of the protons, *θ* is the flip angle. Longitudinal relaxation rate $R_{1}\left(t\right)$ (where $R_{1}$ = 1/$T_{1}$) and transverse relaxation rate $R_{2}^{*}(t)\;$ (where $R_{2}^{*}$ = 1/$T_{2}^{*}$) are the time courses of longitudinal and transverse relaxation rates, respectively, here, ${\;e}^{{-TER}_{2}^{*}(t)}\approx 1$.

### Appendix A.2. Signal-to-CA Concentration Calculation

The signal intensity in a voxel, measured from an SPGR $T_{1}$w sequence for a given FA expressed in Equation (A1) can be expressed for the time course of $R_{1}\left(t\right)$ as follows:

(A3) $$R_{1}\left(t\right)=\frac{1}{TR}ln\left(\frac{1-\left(\frac{S\left(t\right)\cos\left(\theta \right)}{M_{0}\sin\left(\theta \right)}\right)}{1-\left(\frac{S\left(t\right)}{M_{0}\sin\left(\theta \right)}\right)}\right)$$

In the limit of fast water exchange, the change in water proton relaxation rate, Δ$R_{1}$, (i.e., Δ$R_{1}\left(t\right)={(R}_{1}(t)-R_{10})$ [s^−1^]) due to CA relaxivity, $r_{1}$ [(mM)^−1^s^−1^], is linearly related to the tissue CA concentration, $C_{t}$ (mM),

(A4) $${R_{1}\left(t\right)=R_{10}{+r}_{1}C}_{t}\left(t\right)\;\to {\Delta R}_{1}\left(t\right)=r_{1}C_{t}(t)$$

where $R_{10}$ is the pre-contrast longitudinal relaxation rate.

### Appendix A.3. Compartmental Tissue Uptake Model (CTUM)

This model assumes no back flux of CA from the interstitium to the plasma compartment; thus, $v_{e}$ is not measurable. Three parameters, $v_{p}$, $F_{p}$, and $K^{trans}$= E ×$F_{p}$, can be estimated using:

(A5) $$C_{t}(t)=C_{p}⊗F_{p}(t)(e^{-\frac{t}{T_{p}}}+E({1-e}^{-\frac{t}{T_{p}}}))$$

where E is the first extraction fraction, E = PS/(($F_{p}$ + PS)), and $T_{p}$ is the mean plasma transit time, $F_{p}$ is the blood plasma perfusion, and PS is the capillary permeability surface area product [5].

### Appendix A.4. Two-Compartment Exchange Model (2CXM)

The 2CXM estimates $F_{p}$, PS, $v_{p}$_,_ and $v_{e}$ [5].

(A6) $$C_{t}(t)=C_{p}\times A⊗F_{p}(t)$$

where A is given by,

(A7) $$A\left(t\right)=e^{-tB_{+}}+E_{-}(e^{-tB_{-}}-e^{-tB_{+}})$$

and $B_{\pm }$ is given by

(A8) $$B_{\pm }=\frac{1}{2}(T_{p}^{-1}+T_{E}^{-1}\pm \sqrt{{\left(T_{p}^{-1}+T_{E}^{-1}\right)}^{2}-{4T}_{E}^{-1}T_{B}^{-1}})$$

(A9) $$E_{-}=\frac{B_{+}-T_{B}^{-1}}{B_{+}-B_{-}},$$

(A10) $$T_{p}=\frac{v_{p}}{PS+F_{p}}\;;T_{E}=\frac{v_{e}}{PS};T_{B}=\frac{v_{p}}{F_{p}}$$
